# Effect of *Platycodon grandiflorus* Polysaccharide on M1 Polarization Induced by Autophagy Degradation of SOCS1/2 Proteins in 3D4/21 Cells

**DOI:** 10.3389/fimmu.2022.934084

**Published:** 2022-06-30

**Authors:** Liping Li, Xufang Chen, Meiyun Lv, Ziqiang Cheng, Fang Liu, Ying Wang, Aiqin Zhou, Jianzhu Liu, Xiaona Zhao

**Affiliations:** ^1^ College of Veterinary Medicine, Shandong Agricultural University, Tai`an, China; ^2^ Qingdao Animal Disease Prevention and Control Center, Qingdao Municipal Bureau of Agriculture and Rural Affairs, Qingdao, China; ^3^ Research Center for Animal Disease Control Engineering, Shandong Agricultural University, Tai`an, China

**Keywords:** *Platycodon grandiflorus* polysaccharide, 3D4/21 cells, autophagy degradation, SOCS1/2 proteins, M1 polarization

## Abstract

M1-polarized macrophages can improve the body’s immune function. This study aimed to explore the mechanism of *Platycodon grandiflorus* polysaccharide (PGPS_t_) degrading SOCS1/2 protein through autophagy and promoting M1 polarization in 3D4/21 cells. Immunoprecipitation, confocal laser scanning microscopy, flow cytometry, and intracellular co-localization were used to detect the expression of related phenotypic proteins and cytokines in M1-polarized cells. The results showed that PGPS_t_ significantly promoted the mRNA expression of IL-6, IL-12, and TNF-α and enhanced the protein expression of IL-6, IL-12, TNF-α, IL-1β, iNOS, CD80, and CD86, indicating that PGPS_t_ promoted M1 polarization in 3D4/21 cells. Next, the effect of the PGPS_t_ autophagy degradation of SOCS1/2 on the M1 polarization of 3D4/21 cells was detected. The results showed that PGPS_t_ significantly downregulated the expression level of SOCS1/2 protein, but had no obvious effect on the mRNA expression level of SOCS1/2, indicating that PGPS_t_ degraded SOCS1/2 protein by activating the lysosome system. Further research found that under the action of 3-MA and BafA1, PGPS_t_ upregulated LC3B II and downregulated SOCS1/2 protein expression, which increased the possibility of LC3B, the key component of autophagy, bridging this connection and degrading SOCS1/2. The interaction between SOCS1/2 and LC3 was identified by indirect immunofluorescence and Co-IP. The results showed that the co-localization percentage of the two proteins increased significantly after PGPS_t_ treatment, and LC3 interacted with SOCS1 and SOCS2. This provides a theoretical basis for the application of PGPS_t_ in the treatment or improvement of diseases related to macrophage polarization by regulating the autophagy level.

## Introduction

A tremendous amount of scientific work has confirmed the medicinal values of traditional Chinese medicine (TCM). One of the representative TCMs is *Platycodon grandiflorus*, which has been used to treat viral diseases for several centuries in China. *P. grandiflorus* (the rhizome of *P. grandiflorus*) are well-known, commonly used TCM preparations. According to anecdotal evidence passed down for over 2,000 years by the practitioners of the TCM system, *P. grandiflorus* can promote health and homeostasis. In recent decades, the investigations of *P. grandiflorus* have focused on its biological activities, including its anti-tumor, hepatoprotective, immunoregulatory, and antioxidant effects. These studies have resulted in the isolation of saponins, flavonoids, anthocyanins, phenolics, and polysaccharides, among other compounds, from the plant (1). Polysaccharide is the main immunologically active substance in PG, which has an obvious immunomodulatory effect. It is the material basis for “strengthening and restoring effects” and enhancing immune function. In order to explore the immune mechanism of the *P. grandiflorus* polysaccharide (PGPS_t_), our research group has identified the polysaccharide structure of PGPS_t_ and evaluated its immune regulation activities on 3D4/21 cells, chicken peritoneal macrophages, and lymphocytes. Studies have found that PGPS_t_ can enhance the phagocytosis and proliferation activity of chicken peritoneal macrophages, induce the expression of costimulatory molecules (CD80 and CD86), promote the secretion of cytokines and the release of NO, and induce macrophage polarization to the M1 type (2). In addition, PGPS_t_ can promote lymphocyte proliferation, increase the ratio of CD4 +/CD8 + subsets, and promote cells to enter the DNA synthesis phase (3). PG, a polysaccharide isolated from *P. grandiflorum*, could induce macrophage activation through the TLR4/NF-κB signaling pathway and activate MAPK and AP-1, which suggested that PG induces nitric oxide (NO) production and the mRNA expression of iNOS in RAW 264.7 cells (4). Yeo Dae Yoon et al. demonstrated that a polysaccharide isolated from *P. grandiflorums* electively activates B cells and macrophages but not T cells (5).

Macrophages have long been considered to be important immune effector cells. Macrophage polarization refers to an estimate of macrophage activation at a given point in space and time. Polarization is not fixed, as macrophages are sufficiently plastic to integrate multiple signals, such as those from microbes, damaged tissues, and the normal tissue environment. Macrophages can acquire distinct morphological and functional properties in different microenvironments. Different inflammatory stimuli can temporarily induce distinct subsets of macrophages with polarized inflammatory phenotypes. The M1 phenotype is characterized by a high capacity to present antigen, high levels of inflammatory cytokine (TNF-α, IL-6) secretion and increased levels of NO production, an enhanced capacity to kill intracellular pathogens and tumor cells, and the promotion of polarized h1 immune responses (6). Polarized macrophages can further affect the local immune response, and coordinate with various factors to regulate pathogenic microbial infection and tumor immunity, and participate in immune regulation (7). It has been reported that the polarization of macrophages toward the M1 type is very important for effective antiviral immune response. In acute and chronic HIV infection, M1 macrophages in parenchymal tissue play an important role in the early antiviral immune response and subsequent recovery response (8). In addition, some scholars have found that polarized M1 porcine alveolar macrophages (PAMs) have a significant inhibitory effect on the replication and proliferation of PRRSV (9).

Autophagy can activate innate immune cells, and the substrate of autophagy can be presented as an antigen to adaptive immune cells to start adaptive immune response. In recent years, studies on a variety of chronic inflammatory disease models have shown that autophagy may participate in the regulation of macrophage polarization. It has been reported that autophagy occurs in many physiological and pathological situations and is a key component of development and differentiation (10). Some studies suggest that autophagy plays a vital role in viral infection and immunosuppression (11, 12).

Autophagy has recently been considered important in macrophage polarization. Autophagy may be involved in the regulation of macrophage polarization by regulating inflammatory response, reactive oxygen species (ROS), and apoptosis (13). Baicalin promotes the transformation of tumor-associated macrophages (TAMs) from M2- to M1-like phenotypes through the autophagy degradation of TRAF2 to exert anti-tumor effects (14). USP19 inhibits inflammation and promotes M2-like macrophage polarization by regulating NLRP3 function through autophagy (15). The inhibition of USP14 can promote the autophagy of M1-like macrophages and alleviate sepsis caused by CLP (16). It has been found that the IFN-γ/JAK-STAT1 pathway is a key component of the macrophage M1 polarization regulator. The SOCS protein family is a very important regulator of macrophage polarization (17). The expression of STAT, a key protein in the polarization pathway, is affected by the cytokine signal transduction inhibitor SOCS protein (18). For M1 macrophages, SOCS1 protein reduces the secretion of some pro-inflammatory mediators in M1 macrophages (19). After SOCS1 protein expression was downregulated, the proportion of M1 type cells increased significantly, while the proportion of M2 cells did not change significantly. SOCS2 protein knockout macrophages highly express M1 markers (20). In addition, studies have shown that SOCS2 protein can interact with LC3 protein and promote the differentiation of astrocytes through the autophagy degradation of SOCS2 protein (21). These results provide a partial basis for the autophagy degradation of SOCS protein.

In this study, co-immunoprecipitation, laser confocal, flow cytometry, and intracellular co-localization techniques were used to detect the changes in the expression of related phenotypic proteins and cytokines in M1-polarized cells and to determine the degree of polarization of 3D4/21 cells induced by PGPS_t_. The effect of PGPS_t_-induced autophagy degradation of SOCS1/2 on M1 polarization in 3D4/21 cells was studied to clarify the relationship between autophagy and polarization. The co-localization of SOCS1/2 with autophagosomes and autolysosomes was observed. The interaction between SOCS1/2 and LC3 was identified by Co-IP to verify that SOCS1/2 autophagy degradation was mediated by LC3 protein. The purpose of this study was to explore the mechanism of PGPS_t_ inducing the autophagy degradation of SOCS1/2 protein and promoting the M1 polarization of 3D4/21 cells and to provide a theoretical basis for the application of PGPS_t_ in the prevention and treatment of viral infection diseases.

## Materials and Methods

### Reagents and Antibodies

Total PGPS_t_ was prepared in our laboratory, and the polysaccharide content of PGPS_t_ was 76.76%. The information on the extraction methods and structure identification of the polysaccharide has been determined in our previous study (2).

Recombinant porcine interferon-gamma (IFN-𝛾) (985-PI-050) was purchased from R&D Systems (Minneapolis, MN, USA). Lipopolysaccharide (LPS) (L8880) was purchased from Solarbio (Beijing, China). MG-132, chloroquine (CQ), 3-methyladenine (3-MA), and bafilomycin A1 (Baf A1) were delivered by Med Chem Express (Shanghai, China). The Hoechst 33342 kit (C1026) and Nitric Oxide Synthase (iNOS) Assay kit (S0025) were provided by Beyotime Institute of Biotechnology (Haimen, China). The modified RPMI-1640 medium was purchased from Gibco (Shanghai, China). Penicillin-Streptomycin Amphotericin B (03-033-1B/C) and Certified FBS (04-001-1ACS) were purchased from Biological Industries (Kibbutz Beit Haemek, Israel).

Anti-LC3B (ab229327) and SOCS1 (ab9870) were purchased from Abcam (Shanghai, China). Anti-SOCS2 (#2779) and anti-LC3B (#83506) were obtained from Cell Signaling Technology (Shanghai, China). Anti-SOCS3 (14025-1-AP), anti-CD80 (66406-1-lg), anti-α-tubulin (66031-1-lg), anti-SQSTM1/p62 (18420-1-AP), anti-GAPDH (60004-1-lg), HRP-conjugated Affinipure Rabbit Anti-Goat IgG (H+L) (SA00001-4), HRP-conjugated Affinipure Goat Anti-Mouse IgG (H+L) (SA00001-1), HRP-conjugated Affinipure Goat Anti-Rabbit IgG (H+L) (SA00001-2), and CoraLite488-conjugated Affinipure Goat Anti-Mouse IgG (H+L) (SA00013-1) were purchased from Proteintech (Wuhan, China). Cy3-conjugated Goat Anti-rabbit IgG (H+L) (GB21303) was purchased from Servicebio (Wuhan, China). Anti-CD86 (abs120515) was purchased from Absin (Shanghai, China). IFkine™ Red Donkey Anti-Goat IgG (A24431) and IFkine™ Green Donkey Anti-Rabbit IgG (A24221) was provided by Abbkine (Shanghai, China).

### Cell Culture

Porcine alveolar macrophage cell line 3D4/21 cells were obtained from iCell Bioscience (Shanghai, China) and cultured in the modified RPMI-1640 medium adjusted to contain with 2 mM L-glutamine, 1.5 g/L sodium bicarbonate, 4.5 g/L glucose, 10 mM HEPES, 1.0 mM sodium pyruvate supplemented with 0.1 mM nonessential amino acids, 10% fetal bovine serum, and 1% Penicillin-Streptomycin Amphotericin B at 37°C in 5% CO_2_ atmosphere.

### Experimental Treatments

To obtain differentiated macrophages, 3D4/21 cells were stimulated with LPS (150 ng/ml) and IFN-γ (50 g/ml), to promote M1 polarization when grown to 70%–80% confluence in six-well plates; the dosage refers to the study of Wang et al. (22). Then, PGPS_t_ (100 μg/ml) was added to the tested wells; the dosage refers to our previous study (23). To inhibit autophagy, cells were pretreated for 8 h with 3-MA (100 μM) and BafA1 (60 nM), respectively, before PGPS_t_. After different treatments, cells or supernatants were harvested for analyses. In order to study the degradation pathway of SOCS protein, MG-132 (100 nM) and CQ (20 μM) were respectively used in the experiment.

### ELISA Analysis

At indicated time points, the concentration of IL-6, IL-12, TNF-α, and IL-1β in supernatants were measured by ELISA according to manufacturer’s instruction (Porcine IL-6 ELISA Kit, Porcine IL-12 ELISA Kit, Porcine TNF-α ELISA Kit, Porcine IL-1β ELISA Kit, Shanghai, China).

### Real-Time Fluorescence Quantitative Polymerase Chain Reaction

Total RNA was extracted using the RNAiso Plus reagent(Takara, Dalian, China), and then genomic DNA Contaminated gDNA was removed as follows: 2 μL (1 μg) total RNA, 1 μL gDNA Eraser, 2 μL 5× gDNA Eraser Buffer, 5 μL RNase free water were mixed in PCR tube, the reaction was incubated at 42°C for 2 min. Afterwards, 1 μL PrimeScript RT Enzyme Mix I, 1 μL RT Primer Mix, 4 μL 5× PrimeScript Buffer 2, 4 μL RNase free water were added to the tube. The mixture was incubated at 37°C for 15 min, 85°C for 5 s. The cDNA was stored at −20°C for later use. was removed and reverse-transcribed in a BIO-RAD Mastercycler (BIO-RAD, California, USA), using a PrimeScript™ RT reagent Kit (Takara, Dalian, China) with gDNA Eraser according to the manufacturers’ instructions. PCR was performed in triplicate in a LightCycler^®^96 system(Roche Diagnostics GmbH, Mannheim, Germany). The sequences of the specific primers are shown in **Table 1** and were purchased from Takara. After using the threshold cycle value to normalize the expression changes of the gene of interest to the housekeeping gene GAPDH, the folding changes are calculated.

**Table 1:** Primer sequences for real-time PCR analysis.

Gene	Primer	
TNF-α	Forward:	5’-AGAGCATGATCCGAGACGTG -3’
	Reverse:	5’-CAGTAGGCAGAAGAGCGTGGT -3’
IL-6	Forward:	5’-AATAAGGGAAATGTCGAGGCTGT -3’
	Reverse:	5’-GGCATTTGTGGTGGGGTTAG -3’
IL-12	Forward:	5’-TCATCAGGGACATCATCAAACC -3’
	Reverse:	5’-GAACACCAAACATCAGGGAAAAG -3’
SOCS1	Forward:	5’-CCGTCCTCCGCGATTACTTGA -3’
	Reverse:	5’-CCTCCAACCCACATGGTTCCAA -3’
SOCS2	Forward:	5’-GGCACCGTTCACCTCTATCTG -3’
	Reverse:	5’-TAGTCTTGTTGGTAAAGGCAGTCC -3’
GAPDH	Forward:	5’-CACTGGTGTCTTCACGACCAT -3’
	Reverse:	5’-TTCACGCCCATCACAAACA -3’

### Protein Isolation and Western Blotting

After different treatments, cells were lysed with the RIPA buffer containing a protease inhibitor cocktail for protein analysis. Then, protein concentration was measured by the BCA Protein Assay Kit (CWBIO, Beijing, China). Equal amounts of protein samples were size-separated by 12% SDS-PAGE and blotted onto the polyvinylidene fluoride membrane. After blocking, membranes were exposed to antibodies that recognized P62/SQSTM1, LC3B, SOCS1, SOCS2, SOCS3, CD80, and CD86 at 4°Covernight. Then, the membranes were incubated with the corresponding horseradish peroxidase (HRP)–conjugated IgG secondary antibody at room temperature for 1 h. The reactive bands were visualized using the enhanced chemiluminescence detection system and analyzed with Image J software. The relative protein expression levels were normalized to GAPDH or α-tubulin.

### Confocal Microscopy Analysis

The iNOS protein expression levels of the 3D4/21 cells were evaluated by confocal immunofluorescence microscopy according to the iNOS manufacturer’s instruction. In order to observe the morphology of macrophages under different treatment conditions, indirect immunofluorescence was performed using the anti-GAPDH antibody. To elucidate the cytosolic localization of SOCS1 and LC3B, SOCS2, and LC3B, indirect immunofluorescence was performed using the anti-SOCS1 antibody, anti-SOCS2 antibody, and anti-LC3B antibody. Briefly, the 3D4/21 cells were incubated with antibodies overnight at 4°C and then with the corresponding fluorescent dye–conjugated secondary antibodies for 1 h. After washing with PBS, the cells were permeabilized with Hoechst 33342 for 10 min and examined with a Leica TCS SPE confocal microscope (Heidelberg, Baden-Württemberg, Germany).

### Co-Immunoprecipitation

Cell samples were lysed and centrifuged. The supernatant was added to 100 μl of Protein A+G agarose beads and rotated at 4°C for 1 h to remove non-specific binding proteins for further use. The LC3B antibody and corresponding amount of normal IgG were diluted according to the instructions, and 100 μl of Protein A+G agarose beads were added, respectively, rotated for 2 h at 4°C, and then centrifuged for 5 min at 1,000 g and 4°C. The prepared supernatant was added to Protein A+G agarose beads conjugated with antibody or normal IgG, and rotated overnight at 4°C, to bind the protein to the antibody. After removing the supernatant, the supernatant was washed with PBS four times, 1 ml each time. The eluted protein was added to 100 µL 1× loading buffer, boiled for 5 min, and centrifuged for 5 min at 1,000 g, and the supernatant was taken for Western blot detection.

### Statistical Analysis

All data were obtained from three repeated experiments and expressed as mean ± standard deviation (SD). All statistical calculations were performed using SPSS 24.0 software (IBM, Armonk, NY, USA). Differences between groups were analyzed using one-way analysis of variance (ANOVA). The levels of significance were as follows: *P*< 0.05 is signified by *; *P*< 0.01 is signified by **.

## Results and Discussion

### Morphological Changes of M1 Polarization in 3D4/21 Cells

**Figure 1:**
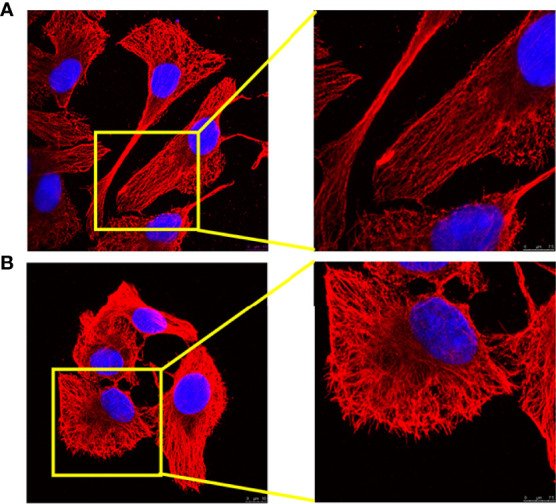
Analysis of 3D4/21-cell morphology under different activation methods. **(A)** Morphology of (M0) 3D4/21 cells incubated in RPMI-1640 for 24 h. **(B)** The morphology of (M1) 3D4/21 cells treated with IFN-γ/LPS was examined by confocal microscopy. M0-type 3D4/21 cells were a non-polarized control group.

After stimulation with LPS/IFN-γ for 24 h, the morphology of 3D4/21 cells changed. Compared with the control group (M0), the nucleus of 3D4/21 cells became larger after polarization, the nucleus became irregular, the cell outline became rounded, and the tubulin became disorderly (**Figure 1**).

Macrophages are stimulated by different environmental factors to differentiate into different phenotypes that can adapt to the internal environment. Macrophages stimulated by IFN-γ and LPS polarize toward the classically activated M1 phenotype macrophages and can trigger an inflammatory response and kill pathogens in the cell (24). Most viruses with monocyte tropism, such as human immunodeficiency virus (HIV), respiratory syncytial virus (RSV), and severe acute respiratory syndrome virus (SARS), may affect macrophages after infection and in turn cause immune suppression of the host, which reduces the body’s immune system’s ability to recognize and eliminate the virus, leading to serious secondary infections (25–27). Tumor-secreted Pros1 inhibits macrophage M1 polarization to reduce antitumor immune response (7, 7). It is imperative to activate macrophages to polarize M1 in diseases such as viral infections, immunosuppression, and tumors. In this study, an *in vitro* M1 polarization model of 3D4/21 cells was established.

In recent years, with the deepening understanding of the function of macrophages, some studies have begun to explore their immune activity from the effect of polysaccharides on the polarization of macrophages.

### Changes in the Expression of M1 Genes in Stimulated 3D4/21 Cells

**Figure 2:**
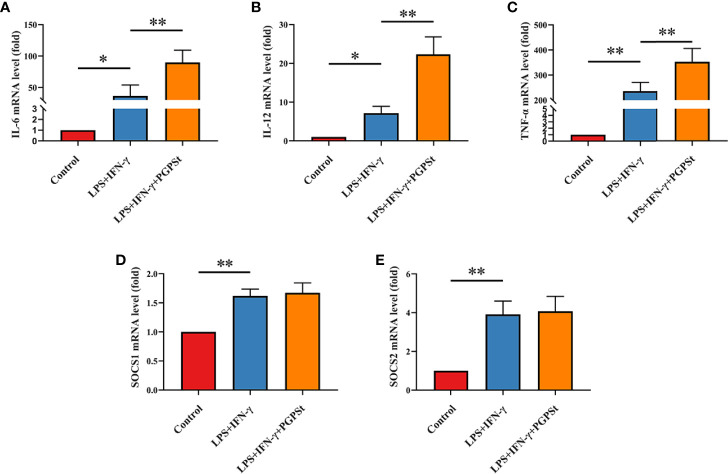
qRT-PCR analysis to determine 3D4/21 cell polarization. After treatment with various reagents, the mRNA levels of M1-type 3D4/21 cells were monitored by qRT-PCR. After incubation for 24 h, these images showed the expression of polarization markers in M1-type 3D4/21 cells, such as **(A)** IL-6, **(B)** IL-12, and **(C)** TNF-α. The expression of regulatory factors **(D)** SOCS1 and **(E)** SOCS2 in the polarization pathway of M1-type 3D4/21 cells. Means ± SD are indicated (n = 3). * indicates *P* < 0.05; ** indicates *P* < 0.01.

In order to confirm the effectiveness of the selected treatment for inducing M1 polarization, qRT-PCR was used to detect the expression of important M1 biomarkers. It is noteworthy that the expression of IL-6, IL-12, and TNF-α in the LPS/IFN-γ treatment group was significantly higher than that in the control group. The mRNA expression of PGPS_t_ was significantly higher than that in LPS/IFN-γ treatment group (**Figures 2A–C**). As shown in **Figures 2D, E**, LPS/IFN-γ can also enhance the mRNA expression level of SOCS1/2, but PGPS_t_ seems to have no effect. These results indicated that LPS/IFN-γ induced the macrophage transformation into the M1 phenotype, in accordance with the ones found in the roots of *Actinidia eriantha* (28).

**Figure 3:**
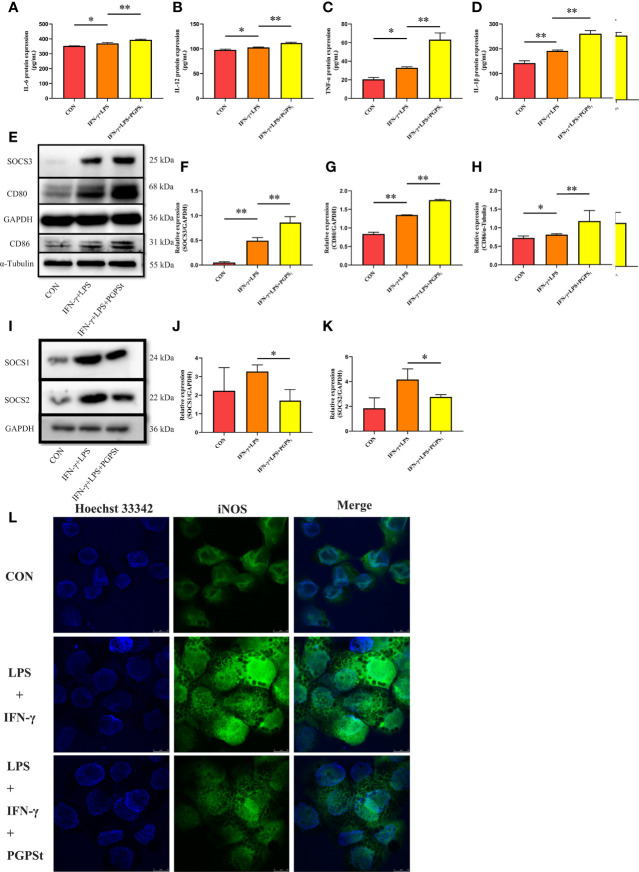
Expression changes of M1 marker protein in 3D4/21 cells treated with LPS/IFN-γ or PGPS_t_ for 24 h. **(A–D)** The expression of M1 marker proteins in different activated 3D4/21 cells was analyzed by the ELISA kit. **(E–H)** 24 h after stimulation, Western blot analysis of M1 marker protein expression in 3D4/21 cells; **(I–K)** Western blot analysis of protein expression regulated by the polarization pathway in 3D4/21 cells. **(L)** Approximately 24 h after stimulation, the proportion of cells expressing iNOS (the marker of M1-type 3D4/21 cells) was monitored by a laser confocal microscope. Means ± SD are indicated (n = 3). * indicates *P* < 0.05; ** indicates *P* < 0.01.

Inducible nitric oxide synthase (iNOS) is an important factor expressed by M1 macrophages, which catalyzes the production of nitric oxide (NO) from the L-arginine substrate. IL-6, IL-12, TNF-α, IL-1β, and iNOS are the markers of M1 macrophages (1). Therefore, the expression levels of these cytokines can be used to evaluate the activation of macrophages. As shown in **Figures 3A–D, L**, compared with the control group, the LPS/IFN-γ treatment group significantly increased the expression levels of IL-6, IL-12, TNF-α, IL-1β, and iNOS. Importantly, the expression of these markers in the PGPS_t_ group was significantly increased compared to the LPS/IFN-γ treatment group alone, which confirmed that PGPS_t_ could promote the production of IL-6, IL-12, TNF-α, IL-1β, and iNOS. An interesting phenomenon was discovered here, that is, PGPS_t_ could downregulate the protein level rather than the mRNA level of polarization pathway inhibitor SOCS1/2. This indicates that PGPS_t_ induces the degradation of SOCS1/2 proteins.

CD80 and CD86 are expressed in activated B and T lymphocytes, macrophages, peripheral blood mononuclear cells, and dendritic cells, which are essential membrane antigens for macrophage activation. In addition, CD80 is the most robust phenotypic marker for human MΦ (IFN-γ) (29). In **Figures 3E, G, H**, the expression levels of CD80 and CD86 in the PGPS_t_ group were significantly higher than those in other groups. Furthermore, the expression of SOCS3, which inhibited M2 polarization, was increased by PGPS_t_ (**Figures 3E, F**). These results further proved that PGPS_t_ could promote LPS/IFN-γ-induced macrophage transformation to the M1 phenotype.

SOCS family is a negative regulator associated with CNS immune inflammatory response. For M1 macrophages, SOCS1 protein attenuates the secretion of certain proinflammatory mediators in M1 macrophages (19). After the downregulation of SOCS1 protein expression, the proportion of M1-type cells increased significantly, while the proportion of M2-type cells did not change significantly; SOCS2 protein knockout macrophages highly expressed M1-type markers (20). SOCS1/2 is likely to become a rational target for therapeutic regimens against various immune diseases that occur on the basis of the overshooting of the immune system, such as immunodeficiency and immunosuppressive diseases. An interesting phenomenon can be observed here; LPS/IFN-γ could increase the expression level of SOCS1/2 protein. However, PGPS_t_ reduced the protein level of SOCS1/2 (**Figures 3I–K**). The inhibitor of cytokine signaling (SOCS) proteins act as feedback inhibitors in the JAK/STAT signaling pathway, which can terminate innate and adaptive immune responses (18). The results indicated that PGPS_t_ promoted LPS/IFN-γ-induced M1 polarization by reducing the inhibitory protein SOCS1/2 in the JAK/STAT signal transduction pathway of M1 polarization.

### SOCS1/2 Was Degraded Through the Lysosomal Pathway

**Figure 4:**
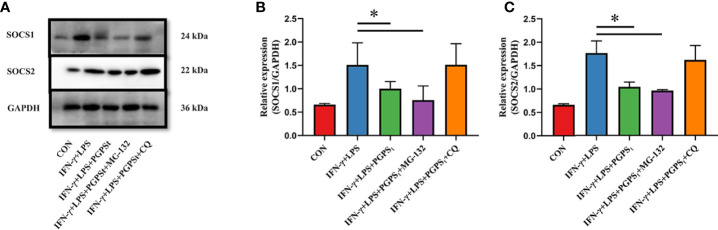
Effects of MG-132 and CQ on SOCS1 and SOCS2 expression in M1-type 3D4/21 cells. **(A–C)** Western blot analysis of the expression of SOCS1 and SOCS2 proteins in M1-type 3D4/21 cell polarization pathway regulators. Means ± SD are indicated (n = 3). * indicates *P* < 0.05.

To explore the degradation pathway of SOCS1/2, the classical proteasome pathway inhibitor MG-132 and lysosome inhibitor CQ were used. As shown in **Figures 4A–C**, PGPS_t_ decreased the expression level of SOCS1/2 proteins. When treated with MG-132, PGPS_t_ can still reduce the expression of SOCS1/2. Conversely, CQ prevented the effect of PGPS_t_ on SOCS1/2. These results indicated that PGPS_t_ could degrade SOCS1/2 proteins by activating the lysosomal pathway. Studies have shown that SOCS2 protein can interact with LC3 protein to degrade SOCS2 protein through autophagy to promote the differentiation of astrocytes (21). Therefore, we speculate that the reduction of SOCS1/2 protein is related to autophagy.

### Regulation of Autophagy by PGPS_t_ Affected the Expression of SOCS1/2

**Figure 5:**
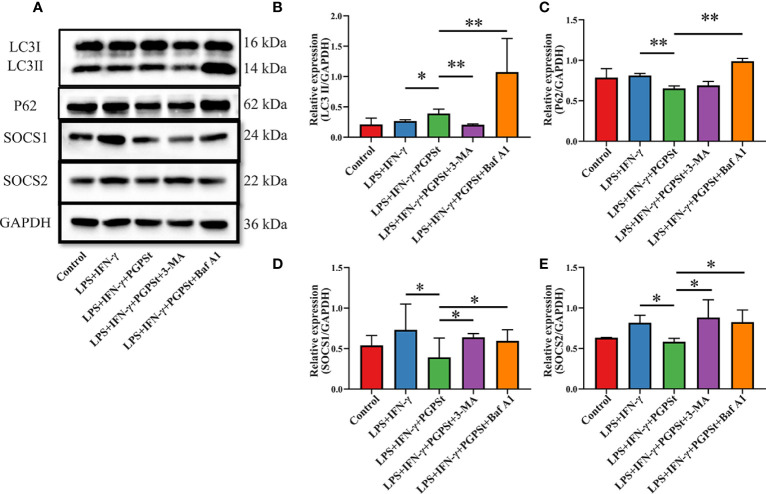
Effect of PGPS_t_ on SOCS1/2 expression through autophagy regulation. **(A)** Western blot analysis of LC3, P62, and SOCS1/2 in 3D4/21 cells in the presence or absence of 3-MA or bafilomycin A1; **(B)** Western blot analysis of LC3II/GAPDH expression; **(C)** Western blot analysis of P62/GAPDH expression; **(D)** Western blot analysis of SOCS1/GAPDH expression; **(E)** Western blot analysis of SOCS2/GAPDH expression. Means ± SD are indicated (n = 3). * indicates *P* < 0.05; ** indicates *P* < 0.01.

The above experimental results mentioned that PGPS_t_ degraded SOCS1/2 proteins by activating the lysosomal pathway, which indicates that autophagy might be involved in this process. It can be seen from **Figures 5A–C** that PGPS_t_ significantly increased the content of intracellular LC3B II and decreased the content of P62. With the effect of the autophagy inhibitor 3-MA, PGPS_t_ promoted the significant reduction of LC3B II and blocked the downregulation of SOCS1/2 protein by PGPS_t_. In addition, with the effect of BafA1, PGPS_t_ promoted the accumulation of LC3B II and P62, and the autophagy flow was blocked. The SOCS1/2 protein is no longer downregulated by PGPS_t_ (**Figures 5D, E**). It can be reasonably assumed that the loss of SOCS1 and SOCS2 on macrophages was due to the enhanced autophagy induced by PGPS_t_. The results showed that under the condition of smooth autophagy, PGPS_t_ upregulated LC3B II and downregulated SOCS1/2 protein expression, which increased the possibility of LC3B, the key component of autophagy, bridging this connection and degrading SOCS1/2.

The previous observations raise fundamental questions: what is the relationship between autophagic machinery and SOCS1/2 degradation? If a relationship exists, what is the functional role of this interaction? The questions indicate a new research field for further investigation of these processes.

### Interaction Between LC3B and SOCS1, LC3B and SOCS2

The above results indicated that PGPS_t_ downregulated SOCS1/2 and increased the possibility that LC3B, the key component of autophagy, might bridge this connection and degrade SOCS1/2. To verify this possibility, the interaction between LC3 and SOCS1/2 was investigated by indirect immunofluorescence and co-immunoprecipitation experiments.

**Figure 6:**
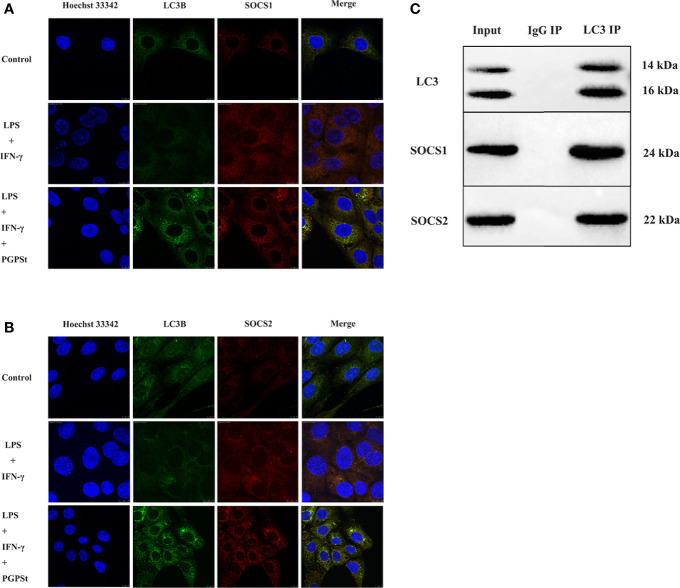
The confocal and co-IP confirmed the co-localization between LC3 and SOCS1, LC3, and SOCS2 in 3D4/21 cells. **(A)** Co-location of SOCS1 and LC3. **(B)** Co-location of SOCS2 and LC3. **(C)** Protein interactions between SOCS1 and LC3, SOCS2, and LC3.

Compared with the control group, the co-localization percentage of the two proteins increased significantly after adding PGPS_t_ (**Figures 6A, B**). The resolution limit of optical microscope means that the physical size and position of small objects in a two-dimensional space or even three-dimensional space are uncertain. The relationship between the two proteins cannot be proved by a single immunofluorescence test. Therefore, the two proteins were detected by immunoprecipitation.

NAs shown in **Figure 6C**, the results showed that LC3, SOCS1, and SOCS2 could be normally detected in the input group, indicating that the expression of three proteins in the cell lysate was normal. The LC3 antibody beads IP complex can detect LC3 expression, while IgG antibody beads IP complex cannot detect LC3 expression, indicating that the LC3 antibody can specifically bind to LC3 protein. The expressions of SOCS1 and SOCS2 could be detected in the LC3 antibody bead IP complex, while the expressions of SOCS1 and SOCS2 were not detected in the IgG bead IP complex, indicating that LC3 might interact with SOCS1 and SOCS2. These data suggest that PGPS_t_ regulated SOCS1/2 protein through the interaction between LC3B and SOCS1/2 protein and that PGPS_t_ was mediated by autophagy in macrophage polarization to M1.

Autophagy participates in the pathophysiological process of many diseases. Through the study of chronic inflammatory disease models, the effect of autophagy on the polarization of macrophages has gradually been confirmed, but under different disease states, the effect of autophagy on the polarization of macrophages is different. Impaired macrophage autophagy promotes proinflammatory macrophage polarization (30). Autophagy promotes M2 macrophage through upregulating ID3 (31). Increased autophagy induces macrophage polarization into M1 with an elevated CD11c population and the gene expressions of proinflammatory cytokines to impair cutaneous wound healing (13).

In view of the important role of macrophages in a variety of diseases and the influence of autophagy on the polarization of macrophages, the research and development of Chinese medicine ingredients to regulate autophagy activity and thereby change the functional state of macrophages is expected to provide new strategies and new ideas for the treatment of related diseases.

In summary, PGPS_t_ promotes the M1 polarization of 3D4/21 cells and enhances the immunoregulatory activity by increasing the autophagy level. As a macromolecular active substance, PGPS_t_ has a complex structure that plays a huge role in immune regulation. Most of the polysaccharides with prominent biological activity are linked by (1→3) glycosidic bonds, with the main chain structure of β (1→3)-D-glucan, which is consistent with the main chain structure of PGPS_t_ (32, 33). The active polysaccharides have a certain molecular weight range. The molecular weight of subpolysaccharides is too large, which is not conducive to their biological activity across the cell membrane into the organism, while the molecular weight is too low, and there is no activity (34). The number-average molecular weight of PGPS_t_ is 1.72 × 10^3^–1.66 × 10^5^ Da, the weight-average molecular weight is 2.05 × 10^3^-2.67 × 10^5^ Da, and the distribution width is 1.19–1.60, indicating that the molecular weight distribution is relatively narrow and the biological activity is better. Polysaccharides are composed of a variety of monosaccharides with more branched chains. Lo et al. (35) studied lentinan (LNT) and found that xylose, mannose, galactose, and their molar ratios are closely related to the biological activity of LNT. Although glucose is an important part of polysaccharides, it has little effect on the biological activity of polysaccharides. PGPS_t_ contains more mannose and xylose, which is consistent with many studies.

## Conclusion

PGPS_t_ promotes the M1 polarization of porcine alveolar macrophages by degrading SOCS1/2 proteins through autophagy and improves the immune function of macrophages.

## Data Availability Statement

The original contributions presented in the study are included in the article/supplementary material. Further inquiries can be directed to the corresponding authors.

## Author Contributions

LL: designed and executed experiments, data curation, data analysis, writing—original draft preparation. XC: data curation, data analysis, writing—original draft preparation. ML, ZC, FL, YW, and AZ: data curation. JL: writing—reviewing and editing, supervision, and project administration. XZ: writing—reviewing and editing, supervision, funding acquisition, and project administration. All authors have revised the manuscript and approved the final version of the paper to be published.

## Funding

This project was supported by the Key Research and Development Program of Shandong Province (Important Science and Technology Innovation Project) (2019JZZY010735), the Natural Science Foundation of Shandong Province, China (ZR2021MC088) and the technology Project of Tai`an science and technology correspondent (2021TPY034).

## Conflict of Interest

The authors declare that the research was conducted in the absence of any commercial or financial relationships that could be construed as a potential conflict of interest.

## Publisher’s Note

All claims expressed in this article are solely those of the authors and do not necessarily represent those of their affiliated organizations, or those of the publisher, the editors and the reviewers. Any product that may be evaluated in this article, or claim that may be made by its manufacturer, is not guaranteed or endorsed by the publisher.
